# Ventilation Modeling of a Hen House with Outdoor Access

**DOI:** 10.3390/ani15152263

**Published:** 2025-08-01

**Authors:** Hojae Yi, Eileen Fabian-Wheeler, Michael Lee Hile, Angela Nguyen, John Michael Cimbala

**Affiliations:** 1 Agricultural and Biological Engineering Department, The Pennsylvania State University, University Park, PA 16802, USA; efw2@psu.edu (E.F.-W.); mlh144@psu.edu (M.L.H.); amn5779@psu.edu (A.N.); 2 Mechanical Engineering Department, The Pennsylvania State University, University Park, PA 16802, USA; jmc6@psu.edu

**Keywords:** poultry, ventilation, animal welfare, model, airflow, CFD

## Abstract

Outdoor access, often referred to as pop holes, is widely used to improve the production and welfare of hens. Such cage-free environments present an opportunity for precision flock management via best environmental control practices. However, outdoor access disrupts the integrity of the indoor environment, including properly planned ventilation. Moreover, complaints exist that hens do not use the holes to access the outdoor environment due to the strong incoming airflow through the outdoor access, as they behave as uncontrolled air inlets in a negative pressure ventilation system. As the egg industry transitions to cage-free systems, there is an urgent need for validated computational fluid dynamics (CFD) models to optimize ventilation strategies that balance animal welfare, environmental control, and production efficiency. We developed and validated CFD models of a cage-free hen house with outdoor access by specifying real-world conditions, including two exhaust fans, sidewall ventilation inlets, wire-meshed pens, outdoor access, and plenum inlets. The simulations of four ventilation scenarios predict the measured air flow velocity with an error of less than 50% for three of the scenarios, and the simulations predict temperature with an error of less than 6% for all scenarios. Plenum-based systems outperformed sidewall systems by up to 136.3 air changes per hour, while positive pressure ventilation effectively mitigated disruptions to outdoor access. We expect that knowledge of improved ventilation strategy will help the egg industry improve the welfare of hens cost-effectively.

## Simple Summary

Cage-free environments with outdoor access offer an opportunity for precision flock management through optimal environmental control practices. However, outdoor access disrupts the integrity of the indoor environment, including the properly planned ventilation. We aimed to develop and validate a CFD model of a cage-free hen house with outdoor access by specifying the real-world conditions and then using mathematical principles of airflow and heat transfer to simulate ventilation performance, leveraging the previous efforts. Computational fluid dynamics models of four different ventilation scenarios have been developed for the Penn State Poultry Education and Research Center (PERC) research room, which features two exhaust fans, sidewall ventilation inlets, wire-meshed pens, outdoor access, and plenum inlets. The simulations of four ventilation scenarios accurately predict the measured air flow velocity with an error of less than 50% for three of the scenarios, and the simulations predict temperature with an error of less than 6% for all scenarios. With a validated research room ventilation model, we can further examine different ventilation strategies to identify those that provide suitable thermal environments with minimal disruptive air-flow patterns. We expect that knowledge of an improved ventilation strategy will help the egg industry improve the welfare of hens cost-effectively.

## 1. Introduction

Precision layer hen house management techniques explore new methods for optimizing modern ventilation systems, or environmental control, i.e., heating, cooling, and air quality, to create an ideal environment for the hens. Some best practices for poultry environmental control systems will also mitigate disease-spreading airborne pathogens. However, it is not straightforward to predict the impacts of varying cage-free environmental settings, e.g., floor-raised, aviary-type, or convertible settings, on the production, comfort, health, and overall well-being of hens. Furthermore, it is challenging to experimentally measure an airflow pattern under varying operational conditions and configurations of exhaust fans, sidewall ventilation openings, bird population density, and other factors. This challenge can be addressed with a validated computational model that can simulate realistic hen house ventilation by modifying geometry and appropriate boundary conditions.

The use of computational fluid dynamics (CFD) in agricultural building modeling has seen significant advancements in recent years. Cook et al. [1] utilized CFD to model Passive Downdraught Evaporative Cooling. Li and Nielsen [2] discussed the challenges of CFD in ventilation research, focusing on turbulence modeling, numerical approximation, and boundary conditions relevant to building ventilation. Valenti et al. [3] employed CFD modeling to enhance the efficacy of heat treatment for insect pest control in a flour mill, aiming to improve building performance and the localization of fan heaters. For poultry house ventilation systems, Blanes-Vidal et al. [4], Seo et al. [5], and Küçüktopcu et al. [6] utilized CFD to predict the environment of broiler houses in different seasons.

Recent advances in CFD modeling have significantly expanded the scope and accuracy of agricultural ventilation research, though notable challenges persist in field measurement and validation approaches. Küçüktopçu et al. [7] provided a comprehensive review highlighting that CFD applications in poultry production have evolved to address five main areas: inlet and fan configuration, ventilation system design, air temperature-humidity distribution, airflow distribution, and particle matter and gas emission, while identifying that the most commonly used turbulence models remain the standard k-ε, RNG k-ε, and realizable k-ε models. However, their analysis revealed persistent challenges in obtaining accurate field measurements due to the inherent variability and instability of field conditions, which require significant investments in time and labor. Consequently, some researchers have relied on alternative approaches, such as wind tunnel tests and scale model simulations, rather than direct field validation. Hu et al. [8] advanced ventilation system design by developing a positive-and-negative-pressure-combined ventilation system for multilayer-caged laying hen houses, demonstrating improved environmental control with reduced temperature differences and enhanced air velocity distribution. Similarly, Choi et al. [9] developed dynamic CFD models for tunnel ventilation in broiler houses. Both studies acknowledge discrepancies between simulated and measured airflow and temperature distributions, which are attributed to potential sensor accuracy issues, unaccounted-for equipment and infiltration effects, and inadequate representation of initial field conditions.

Beyond poultry house, Sousa et al. [10] demonstrated the application of CFD in investigating convective heat transfer in dairy cattle during grazing, revealing that animal orientation relative to airflow significantly influences heat loss. Although their study relied on simplified geometric models and had limited field validation, this study highlights the versatility of CFD in environmental conditions relevant to livestock. While these studies collectively demonstrate the growing sophistication of CFD applications in agricultural building ventilation, they also highlight a persistent gap between computational predictions and field realities, emphasizing the critical need for more robust validation methodologies that can better capture the complex, dynamic nature of real-world agricultural environments.

We simulated the environment of a poultry building by leveraging our previous projects that demonstrated the potential of computational fluid dynamics modeling of poultry house ventilation systems to provide comfortable conditions and contain disease vectors in floor-raised cage-free hen environments [11,12,13,14]. Poultry house ventilation CFD modeling can accurately reflect real-world conditions through the mathematical principles of airflow and heat transfer, simulating the performance of the ventilation system.

Our most recent modeling work [11,12,13] was successfully verified, giving us confidence in the simulation results, but was not validated by comparing the prediction with the actual commercial hen house ventilation data. Our current project investigated two issues related to a cage-free hen environment. First, during our previous project, anecdotal observations by us and industry partners noted the disruption of the indoor environment’s ventilation airflow patterns when hens were given outdoor access through small floor-level doors, which may be referred to as pop holes [15]. Egg producers reported that hens did not use these holes to access the outdoor environment, possibly due to strong incoming airflow through these unregulated ventilation inlets, which also disrupted the planned ventilation. Secondly, many hens are housed in multi-tier housing, such as aviaries, enriched, or convertible housing, and these environments could benefit from an assessment of ventilation options.

Leveraging our knowledge, this study aimed to develop a validated CFD model of a cage-free poultry facility with varying ventilation configurations including outdoor access doors and the addition of positive pressure ventilation. To achieve this goal, this study comprises three main priorities: ensuring ventilation model accuracy via validation; modeling ventilation when hen outdoor access is provided; and comparing the consequences of different ventilation strategies, i.e., positive, neutral, or negative pressure ventilation schemes. The long-term benefit of this project to the egg industry will be the provision of recommendations for cost-effective, welfare-friendly hen housing environments. The ability to predict thermal environments and ventilation airflow velocity patterns can document comfortable conditions for the birds. This paper reports the results of CFD modeling validation of one large research room at Penn State’s Poultry Education and Research Center.

## 2. Method and Materials

### 2.1. Penn State Poultry Education and Research Center

**Figure 1:**
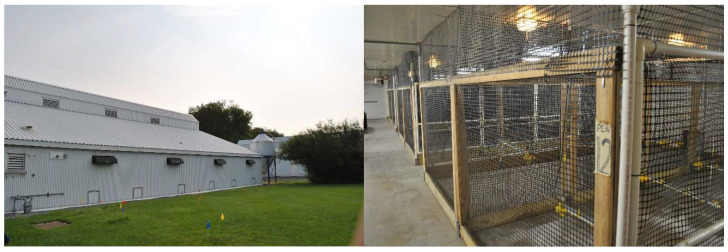
Photographs of Penn State Poultry Education and Research Center Research room.

The CFD model is based on dimensions and measurements of a research room at Penn State’s Poultry Education and Research Center (PERC). The modeled PERC research room includes pop-holes to the exterior (Figure 1 left) and indoor pens (Figure 1 right). The room has twelve pens, paired in three sections per side, supported by a central aisle. Each pen is enclosed with plastic mesh panels, floor to ceiling, to enclose the hens.

The ventilation system can be described as a negative pressure system design featuring two end-wall exhaust fans (variable-speed, 24-inch diameter, with top-hinged shutters and exterior hoods) and traditional sidewall static pressure-controlled inlets positioned near the eaves. These inlets feature an older design with horizontal sliding panel opening via cable system (rather than a hinged design). During warmer weather, more fresh air is supplied through inlets of the same design, located on both sides of a central aisle and an overhead duct. This overhead duct receives air from an insulated plenum separate from the building attic space. Ventilation adjustments are achieved via electronic controller with temperature sensors located near the middle of the room at about human head height. Static pressure difference is automatically monitored via an instrument (Dwyer Magnesense MSX (Dwyer Instruments Inc., Michigan City, IN, USA)) with a flexible tube, located in the research room and the unventilated attic space.

The research room was modified to include outdoor access for the hens via the installation of pop-hole openings in each pen. During the renovation, the overhead plenum space was converted into a positive pressure zone, with one end-wall fan (36 inches in diameter) providing air to the hen room’s central duct assembly. This plenum air supply can operate under either negative or positive pressure, with the latter achieved by installing a fan in the plenum end wall.

To validate the CFD model, we measured the velocity, pressure, and temperature of air inside the PERC facility using four different combinations of ventilation apparatus, which utilized combinations of two exhaust fans, side wall inlets, outdoor access (open or closed), and overhead plenum inlets (Table 1). To track the ventilation conditions and respective results, acronyms of active inlets are used.

### 2.2. Ventilation Scenarios and Boundary Conditions

Four ventilation scenarios were developed to reflect plausible combinations of the status of sidewall ventilation inlets, plenum ventilation inlets, outdoor access, and exhaust fan. These four scenarios are listed in Table 1. It should be noted that the SNO condition is a typical negative pressure ventilation strategy used in commercial hen houses.

The addition of pop-holes for outdoor access is expected to disrupt the proper functioning of the air inlet portion of a negative-pressure ventilation system. The pop-holes will act as an air inlet at bird level, with an air velocity similar to that of air entering through the planned inlet system. There is some speculation among poultry producers that hens are hesitant to move through the high-velocity air to access the outdoor environment provided by the pop-holes. Importantly, air entering through the pop-holes reduces the amount of air entering the planned eave (and/or ceiling) inlets in a poultry house since the ventilation controller will reduce the planned inlet opening area to maintain a setpoint static pressure difference. Overall, air distribution patterns in the hen house will be disrupted. In the PERC research room, the addition of positive pressure capabilities to the central aisle overhead inlets ensures guaranteed fresh inlet air distribution, similar to that desired when pop-holes are closed. These positive pressure inlets also allow the possibility of employing a neutral pressure ventilation approach.

The SNO condition is a common ventilation strategy in commercial hen housing in buildings without outdoor access or when outdoor access is closed. Outdoor access is open in ventilation scenarios 1, 2, and 3 with different combinations of side wall and plenum inlet status. Air velocities and temperature at exhaust fans, side wall inlet vents, plenum inlet vents, plenum positive pressure fan, and outdoor access were measured in the research room and used as boundary conditions of CFD models.

**Figure 2:**
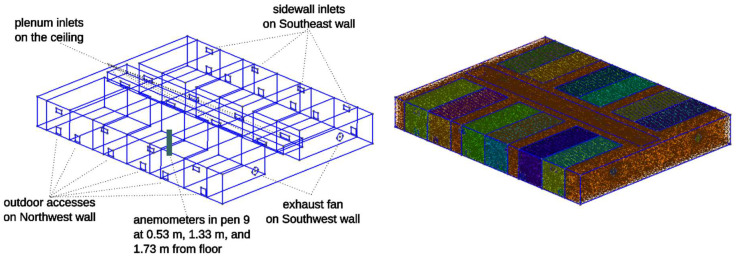
Research room layout (**left**) and volumetric CFD mesh (**right**). Two exhaust fans are represented as two circular holes facing the lower right wall, roughly facing the Southwest direction. Different colors of the meshed volumes (**right**) represent continuous volume enclosed by wire screens. Different colors of the mesh represent pens. A square hole at the bottom of each pen is outdoor access, rectangular openings on the Northwest and Southeast walls are passive ventilation sidewall inlets. Rectangular openings of a box at the center of the henhouse are plenum inlets.

The airspeed and temperatures are measured with an Omni-directional Spherical Probe (Model 6543-2G) (Kanomax USA, Inc., Andover, NJ, USA) connected to a Climomaster Anemometer (Kanomax 6501-OE) (Kanomax USA, Inc., Andover, NJ, USA) and two hot-wire anemometers (Extech Hot Wire Thermo-Anemometer (Industrial electronic Inc., Knoxville, TN, USA)). These anemometers are placed at the center of Pen 9, which is approximately at the center of the research room, and on the west side wall. The heights of the anemometers are 0.53 m, 1.33 m, and 1.73 m from the floor, respectively (Figure 2). The height of these sensors represents bird-level, considering the bird-occupied zone typically extends from floor level to approximately 1.5 m height in commercial facilities [11,16].

The Omni-directional Spherical Probe was placed at 1.13 m from the floor, whereas two hot-wire anemometers were placed at 0.53 m and 1.73 m facing the exhaust fan and side wall, respectively. The comparison between the measured values and the CFD model prediction is made with values at the middle, i.e., 1.13 m from the floor. The air velocity and temperature were measured in Pen 9 and used to validate the simulation results. Air velocity was measured using hot-wire anemometers for room air movement and at inlet openings, and with a vane anemometer for fan air movement.

In the research room CFD model, a no-slip boundary condition was imposed on the walls. Closed outdoor access, side walls, and plenum ventilation inlets were modeled as wall boundary conditions. When open, outdoor access and sidewall ventilation inlets are modeled as passive inlet vents, whereas plenum ventilation inlets were modeled as velocity inlets with 4.6 m/s, based on the validation measurements. For open inlets, temperature was assigned to reflect the measured values ranging from 19.7 °C to 21.7 °C, reflecting outdoor air temperature. Two exhaust fans were modeled as velocity outlets to push air out of the research room, with the measured average air velocity of 3.6 m/s and 3.2 m/s for the south end wall east and west side fans, respectively. The air velocities of exhaust fans were determined with the nine-point measurement on the fan face using the Extech Thermo-Anemometer with a vane probe.

This study was conducted without modeling the presence of birds in the research room, which aligned with our experimental validation conditions where field measurements were taken in an empty facility. This approach enabled direct validation of CFD predictions against measured data, without the need for additional variables, by established CFD validation protocols. The absence of birds allowed for controlled assessment of pure ventilation system performance across different scenarios, isolating the effects of ventilation configurations from biological influences.

However, it is known that bird presence significantly affects ventilation efficiency and air movement patterns through multiple mechanisms, including heat generation that alters thermal gradients and buoyancy-driven flows, physical obstruction that modifies airflow patterns and creates localized turbulence, and resistance to airflow that affects overall air distribution. Such effects can be incorporated with CFD models as shown by Chen et al. [11,12], which successfully modeled individual hens using simplified geometric shapes. As a more simplified approach, porous media modeling was employed by treating animal-occupied zones as porous domains with specified resistance coefficients [17]. This method provides computational efficiency while capturing essential flow resistance effects, making it practical for large-scale facility modeling where individual bird geometry would be computationally prohibitive.

Future applications of our validated CFD framework can readily incorporate these bird modeling approaches to assess the combined effects of ventilation strategies and bird presence on environmental conditions. Such integration would be particularly valuable for optimizing commercial poultry house designs where bird welfare, ventilation efficiency, and energy consumption must be balanced under realistic operational conditions.

### 2.3. Computational Fluid Dynamics Modeling of the Ventilation

The variables of interest for the CFD model and validation include the velocity, pressure, and temperature of air inside the research room. A two-equation turbulence model was used to calculate these domain variables. Specifically, this study used *k-ω* model instead of *k-ω* because of *k-ω* is known to predict more accurately near walls [18] and the effects of walls and pens on the ventilation performance are of main interest in this study.

Fluent implements the *k-ω* model by solving the turbulent kinetic energy transport equation (Equation (1)) and dissipation rate transport equation (Equation (2)) shown below:

Turbulent kinetic energy transport equation:

(1) $$\frac{\partial \left(\rho k\right)}{\partial t}+\frac{\partial \left(\rho u_{j}k\right)}{\partial x_{j}}=\rho P-\beta^{*}\rho \omega k+\frac{\partial }{\partial x_{j}}\left[\left(\mu +\sigma_{k}\frac{\rho k}{\omega }\right)\frac{\partial k}{\partial x_{j}}\right],\;\;\mathrm{with}\;P=\tau_{ij}\frac{\partial u_{i}}{\partial x_{j}}$$

Dissipation rate transport equation

(2) $$\frac{\partial \left(\rho \omega \right)}{\partial t}+\frac{\partial \left(\rho u_{j}\omega \right)}{\partial x_{j}}=\frac{\alpha \omega }{k}\rho P-\beta \rho \omega^{2}+\frac{\partial }{\partial x_{j}}\left[\left(\mu +\sigma_{\omega }\frac{\rho k}{\omega }\right)\frac{\partial \omega }{\partial x_{j}}\right]+\frac{\rho \sigma_{d}}{\omega }\frac{\partial k}{\partial x_{j}}\frac{\partial \omega }{\partial x_{j}}$$

where *ρ* is the density of the fluid, *k* is the turbulent kinetic energy, *ω* is the specific rate of dissipation of the turbulence kinetic energy (*k*) into internal thermal energy, *u* is velocity, *p* is pressure, *μ* is the viscosity, and b and σ are closure coefficients. Appropriate values for the constants were taken from Wilcox [18].

The *k-ω* Shear Stress Transport (SST) turbulence model was chosen for this study, considering that the PERC research room is configured with twelve pens that create multiple walls and compartmentalized volumes. The *k-ω* model has been recognized for its superior accuracy in the viscous sublayer and near-wall regions compared to k-ε models.

Furthermore, research has demonstrated that the SST (Shear Stress Transport) variant of the *k-ω* model is particularly suitable for low-speed, complex indoor environments where adverse pressure gradient conditions are commonly encountered in agricultural buildings with multiple inlets and obstacles [19,20].

In addition, the *k-ω* SST model employs a blending function that automatically switches between *k-ω* formulation near walls and *k-ε* behavior in the free stream [21]. This hybrid approach combines the near-wall accuracy of *k-ω* with the free-stream robustness of *k-ε*, making it particularly well-suited for indoor environments where both wall effects and bulk flow regions are important.

In addition, the heat transfer equation is coupled with the solution of the *k-ω* model equations. The heat transfer equation allows the calculation of the temperature distribution in the PERC research room. It is also possible to couple a mass transfer with the *k-ω* model and heat transfer equations, which allow calculations of airborne particulate matter or chemical species of interest. However, particulate matter and species transfer are beyond the scope of this study and were not included in the CFD model.

### 2.4. Modeling of Pen

The pen of the PPREC hen house is constructed with wire screens with rectangular openings. The pens of the PERC hen house are modeled as a porous jump based on the experimental study on the relationship between wire mesh and pressure drop [22,23,24]. Further, a porous jump is commonly used for modeling a thin, porous geometry where the pressure change is more significant than the thickness [21]. In Fluent, the porous jump model is implemented as a thin membrane with specified pressure drop characteristics. The pressure drop across the porous jump is calculated using the following Equation (3):

(3) $$\Delta p=-\left(\frac{\mu }{\alpha }v+C_{2}\frac{1}{2}\rho v^{2}\right)\Delta m$$

where Δp, μ is the laminar fluid viscosity, α is the permeability of the medium, $C_{2}$ is the pressure-jump coefficient, v is the velocity normal to the porous face, and Δm is the thickness of the porous jump.

## 3. Results and Discussion

### 3.1. PERC Research Room Model Geometry and Mesh

The geometric research room model was constructed and meshed using gmsh v.4.11.1 [25] as shown in Figure 2. The 3D mesh of PERC research room was imported into Ansys (v2023R2) to simulate the airflow, pressure, and temperature distribution. The Ansys Fluent model was assigned with appropriate material properties, i.e., air and wire-mesh of pens, and boundary conditions reflecting different ventilation scenarios, whose details are described in the subsequent section. The pen wire mesh was modeled as a porous jump that impedes airflow based on the size of opening and wire widths [22,23,24]. Boundary conditions include two exhaust fans, outdoor access, eight side wall inlets, and eight plenum.

### 3.2. Simulation Results and Validation

**Figure 3:**
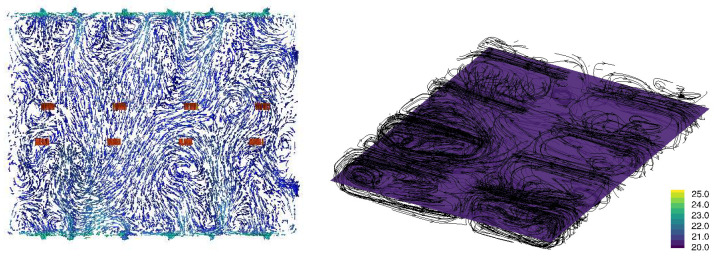
PONE Ventilation condition CFD simulation result: Air flow velocity vectors at the height of 1.13 m from the floor (**left**), temperature at the height of 1.13 m from the floor, and airflow stream patterns (**right**). In the left plot, the blue arrows indicate slower velocity, and the red arrows indicate higher velocity. In the right plot, the streamlines are randomly seeded, and the density of lines does not indicate the intensity of air flow. The legend of the right plot represents temperature in Celsius.

**Figure 4:**
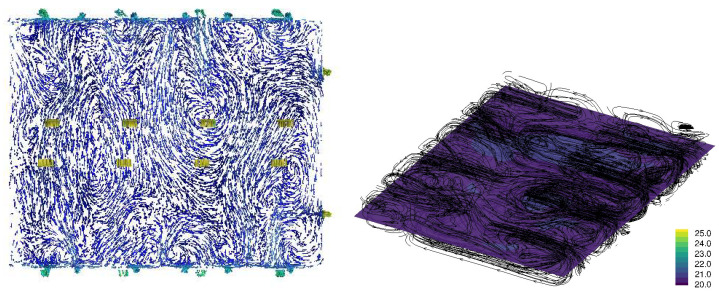
SPO ventilation condition CFD simulation result: Air flow velocity vectors at the height of 1.13 m from the floor (**left**) and the temperature at the height of 1.13 m from the floor and airflow stream patterns (**right**). In the left plot, the blue arrows indicate slower velocity, and red arrows indicate higher velocity. In the right plot, the streamlines are randomly seeded, and the density of lines does not indicate the intensity of air flow. The legend of the right plot represents temperature in Celsius.

**Figure 5:**
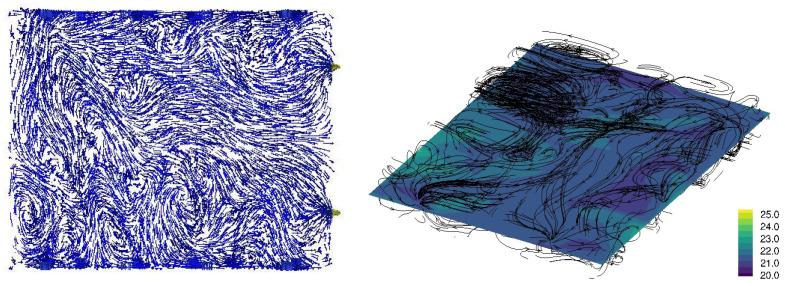
SO ventilation condition CFD simulation result: Air flow velocity vectors at the height of 1.13 m from the floor (**left**) and the temperature at the height of 1.13 m from the floor and airflow stream patterns (**right**). In the left plot, the blue arrows indicate slower velocity, and red arrows indicate higher velocity. In the right plot, the streamlines are randomly seeded, and the density of lines does not indicate the intensity of air flow. The legend of the right plot represents temperature in Celsius.

**Figure 6:**
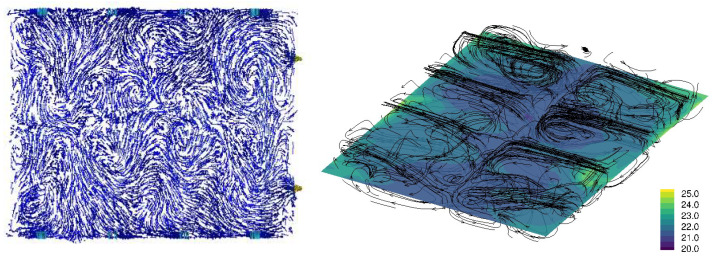
SNO ventilation condition CFD simulation result: Air flow velocity vectors at the height of 1.13 m from the floor (**left**) and the temperature at the height of 1.13 m from the floor and airflow stream patterns (**right**). In the left plot, the blue arrows indicate slower velocities, and the red arrows indicate higher velocities. In the right plot, the streamlines are randomly seeded, and the density of lines does not indicate the intensity of air flow. The legend of the right plot represents temperature in Celsius.

Figure 3, Figure 4, Figure 5 and Figure 6 illustrate airflow vectors, airflow patterns, and temperature of ventilation scenarios 1 to 4, respectively. In Figure 3, Figure 4, Figure 5 and Figure 6, the airflow vectors and temperature values shown are sliced on the *z* plane at the height of 1.13 m from the floor. Table 2 lists measured air flow velocity and temperature values at the center of pen 7, which is 10 m away from the Northeast wall and 1.2 m away from the Southwest wall. Table 2 also lists prediction errors based on the measured values.

Figure 3 illustrates the airflow vectors, airflow patterns, and temperature of the PONE condition. The predicted air velocity is 0.44 m/s and the measured value is 0.7 m/s, resulting in an error of 37.1%. The predicted temperature in pen 9 is 20.4 °C, and the measured temperature was 21.6 °C, resulting in an error of 5.7%. It is also notable that most airflow patterns are contained in each pen, which suggests that even a wire-meshed wall significantly impedes air flow. This is likely due to the relatively low air speeds in the room environment. Figure 4 illustrates the airflow vectors, airflow patterns, and temperature of the ventilation SPO condition. The predicted air velocity is 0.30 m/s and the measured value is 0.36 m/s, resulting in an error of 17.4%. The predicted temperature in pen 9 is 20.4 °C, and the measured temperature is 21.4 °C, resulting in the error of 4.5%. Similar to the SPO condition, airflow patterns are contained in each pen but to a lesser degree, which can be attributed to the opening of the side wall vents.

Figure 5 illustrates the airflow vectors, airflow patterns, and temperature of the SO ventilation condition. The predicted air velocity is 0.17 m/s and the measured value is 0.28 m/s, resulting in an error of 36.5%. The predicted temperature in pen 9 is 22.1 °C, and the measured temperature is 21.1 °C, resulting in an error of 4.8%. Similarly to PO and SPO conditions, airflow patterns are contained in each pen, but this trend is more pronounced in pens further away from exhaust fans.

Figure 6 illustrates the airflow vectors, airflow patterns, and temperature of the SNO ventilation condition. The airflow vectors and temperature shown are sliced on the *z* plane at the height of 1.13 m from the floor. The predicted air velocity is 0.08 m/s and the measured value is 0.31 m/s, resulting in an error of 73.6%. The predicted temperature in pen 9 is 21.7 °C, and the measured temperature is 21.5 °C, resulting in an error of 1.0%. Similarly to PO, SPO, and SNO conditions, airflow patterns are contained in each pen. As previously observed, the air-flow containment is more pronounced in pens further away from exhaust fans.

Regarding the temperature, all four scenarios have similar temperatures ranging from 21.1 °C to 21.6 °C in measurement, and from 20.4 °C to 22.1 °C from CFD simulation, respectively. However, in overall temperature distribution, the SNO condition appears to result in the highest temperature, followed by the SO condition (Figure 5 and Figure 6). Scenarios 1 and 2 appear to result in a lower temperature distribution (Figure 3 and Figure 4) than that of scenarios 3 and 4. This observation seems reasonable because scenarios 1 and 2 involve more airflow with outdoor access opened and plenum inlets running. Overall, temperature prediction errors are less than 8% in all conditions.

Overall, the CFD simulation predicts the measured air flow velocity with less than 40% error for PO condition. Predictions for SPO and SNO conditions are less than 50% error except for the top location in SPO conditions, and the bottom area in SNO conditions, respectively. Prediction of SO conditions is least accurate with approximately 80% errors for top and bottom locations, respectively, and less than 50% error at the middle area. It should be noted that those air velocity prediction errors below 50% for most scenarios and temperature prediction errors below 6% for all scenarios demonstrate successful model validation by previous research reporting comparable magnitudes of errors based on field measurements [7,26].

Notably, the predictions of the middle location are consistently more accurate than those at the top and bottom locations. This may indicate that the omni-directional anemometer measurement is more accurate than directional anemometers. It should also be noted that the SNO condition was implemented with incomplete sealings around outdoor access, which was thought to be a major source of inaccurate air flow velocity predictions. Similarly, the PO conditions were implemented with an incomplete sealing around the sidewall inlets, which improved the prediction accuracy. These results validate the CFD model of Penn State PERC research room ventilation systems. Using the validated model, we can now investigate the effects of changes and structural arrangement of the research room beyond these four ventilation scenarios.

The main goal of adding the plenum ventilation system is to achieve neutral pressure ventilation, which is thought to be able to mitigate wind effects and provide balanced air distribution. To investigate the impact of different ventilation scenarios on the pressure of the PERC research room, the differential pressures of each scenario were measured and predicted. The measured and simulated pressures of the Penn State PERC research room are listed in Table 3. Because of the magnitude of fluctuations of the pressure measurements, the prediction errors are large. However, our model correctly predicts pressure regimes of each ventilation strategy.

Conventional ventilation set up includes exhaust fans running with the side wall openings and without the outdoor access (Scenario 4, SNO), which has negative pressure measurement. The PERC CFD model also predicts negative pressure ventilation. Drawbacks of installing outdoor accesses include the reduction in the airflow and draft from these additional openings. This anecdotal observation is further examined in the following section. In terms of the pressure condition, scenario 3 (SO) indicates that the ventilation operates as a neutral pressure condition as measured, while the PERC CFD model predicts a slight negative pressure ventilation.

To mitigate the detrimental effects of outdoor access on the ventilation, the plenum ventilation system was added to increase the airflow. The measurement and PERC CFD model prediction of Scenario 2 (SPO) indicate that the additional airflow from the plenum inlet creates positive pressure ventilation, which will minimize airborne pathogen entering the animal housing. This positive pressure ventilation is maintained even when the exhaust fans were not running (Scenario 1, PONE).

It should be noted that neutral pressure ventilation is thought to reduce drafts and provides a stable and homogeneous microclimate compared to positive or negative pressure ventilation systems. This will be advantageous in animal housing or plant growth facilities by providing a more stable microclimate. In addition, because neutral ventilation does not need to work against external pressure as much as positive or negative pressure ventilation requires, neutral pressure ventilation systems may consume less energy. Finally, a neutral pressure environment can prevent the spread of airborne pathogens by ensuring that the air does not flow between areas with controlled filtration, especially when combined with a positive pressure antechamber.

On the other hand, neutral pressure systems may offer less control over the direction and speed of airflow compared to positive or negative pressure systems. Without a deliberate pressure differential, there might be a risk of stagnant air pockets forming if the system does not include adequate air circulation mechanisms, which can be exacerbated by additional air-flow impediments such as pens. In addition, neutral pressure systems can be more susceptible to external weather conditions, such as wind, which may disrupt the balance of air pressure inside the facility.

The effect of pens with and without wire-mesh walls on overall airflow and thermal environment

**Figure 7:**
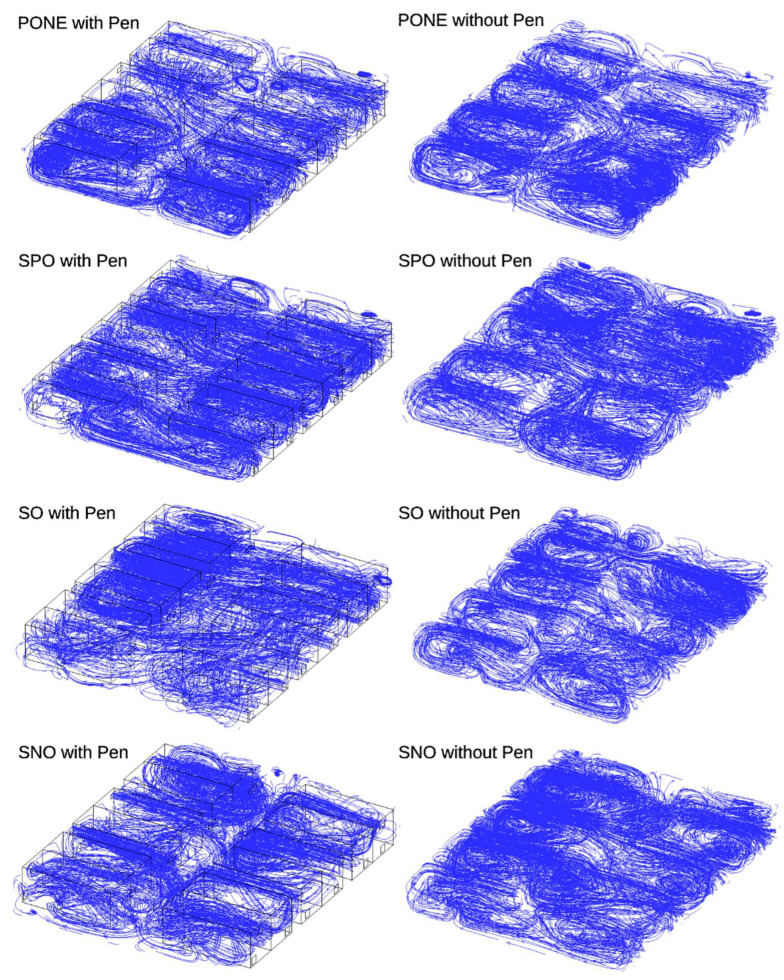
Air flow patterns without pen (**left**) and with pen (**right**).

The CFD simulation investigates four distinct ventilation scenarios, each representing a unique combination of inlet configurations and outdoor access conditions. These scenarios are further subdivided to examine the impact of obstructions, specifically the presence or absence of a pen within the simulated space. The results provide a comprehensive understanding of how different ventilation strategies and environmental factors influence airflow patterns (Figure 7 and Table 2). The presented data showcase predicted values from CFD simulation, offering valuable insights into the airflow dynamics of various ventilation scenarios in a controlled environment.

In Figure 7, flow patterns appear to emerge based on the locations of exhaust fan, side wall ventilation and outdoor access, and plenum ventilation openings and whether they are open, operational, or closed. From the visual observation of left streamline illustrations, the hen house with pen appears to develop isolated airflow either in a pen or in aisles between pens. While there exist clustered air flows in the hen house model without pen, airflows are less confined in the pen or aisle between pens. Such observations can be quantified by computing air flux through the walls of each pen. Based on the air flux, volume flow rates, and Air-Change-per-Hour (ACPH) are estimated (Table 4).

Scenario 1, designated as PONE (Plenum inlet running with Outdoor access open and No Exhaust fan), demonstrates high efficiency in the simulation. In the absence of obstructions from the pen, this configuration predicts a volume flow rate of 30.8 m^3^/s, equivalent to 65.2 × 10^3^ cubic feet per minute (cfm), resulting in 132.3 air changes per hour. This indicates rapid and thorough air circulation within the space. However, the introduction of a pen significantly alters the airflow dynamics, with the simulation predicting a substantial reduction in the volume flow rate to 7.6 m^3^/s (16.0 × 10^3^ cfm), corresponding to only 32.5 air changes per hour. This stark contrast underscores the profound impact that obstructions can have on ventilation efficiency, even in optimal inlet configurations.

Scenario 2, labeled as SPO (Sidewall inlet opened, Plenum inlet running, and Outdoor access opened), emerges as the most efficient configuration in the updated simulation results. Without obstructions from the pen, this scenario achieves the highest volume flow rate of 31.73 m^3^/s (67.2 × 10^3^ cfm), resulting in 136.3 air changes per hour. This slight superiority over Scenario 1 suggests that the combination of side and plenum inlets with open outdoor access may offer optimal air circulation in unobstructed conditions. When a pen is introduced, the flow rate reduces to 13.01 m^3^/s (27.6 × 10^3^ cfm) with 26.8 air changes per hour. Notably, Scenario 2 appears more resilient to the presence of obstructions compared to Scenario 1, as the reduction in flow rate is less severe.

Scenario 3, denoted as SO (Sidewall inlets open and Outdoor access open), represents a shift to sidewall-based ventilation. The CFD model predicts notably lower flow rates for this configuration. Without obstructions, the simulation estimates a volume flow rate of 10.7 m^3^/s (22.7 × 10^3^ cfm), corresponding to 46.0 air changes per hour. When a pen is introduced, the flow rate decreases to 6.2 m^3^/s (13.2 × 10^3^ cfm), maintaining 26.8 air changes per hour. These results suggest that while sidewall inlets are less efficient than plenum-based systems, they may offer more consistent performance in the presence of obstructions.

Scenario 4, represented as SNO (sidewall inlet open and no outdoor access), explores the impact of closing off outdoor access while relying on sidewall ventilation. The CFD simulation predicts the lowest flow rates for this configuration among all scenarios. Without obstructions from a pen, it achieves a volume flow rate of 9.3 m^3^/s (19.6 × 10^3^ cfm) with 39.8 air changes per hour. The presence of a pen reduces the flow rate to 7.2 m^3^/s (15.2 × 10^3^ cfm), resulting in 30.8 air changes per hour. Interestingly, this scenario shows the smallest reduction in flow rate when an obstruction is introduced, suggesting that closed systems might offer more stable, albeit lower, ventilation rates in the presence of internal obstructions.

The CFD simulation results reveal several critical insights into ventilation dynamics. Firstly, they consistently predict that plenum-based inlet configurations (Scenarios 1 and 2) are significantly more effective in generating high volume flow rates and air changes compared to sidewall-based systems (Scenarios 3 and 4). This difference is particularly pronounced in obstruction-free conditions, where plenum-based systems achieve nearly three times the air changes per hour of sidewall systems. Secondly, the simulation underscores the substantial impact of obstructions on airflow patterns. Across all scenarios, the introduction of a pen leads to a marked decrease in volume flow rates and air changes per hour. However, the magnitude of this impact varies considerably between configurations. Plenum-based systems, while more efficient overall, appear more susceptible to performance degradation when obstructions are present. In contrast, sidewall-based systems, though less efficient, demonstrate more consistent performance across obstructed and unobstructed conditions.

The CFD predictions also highlight the role of outdoor access in ventilation efficiency. Comparing Scenarios 3 and 4 reveals that open outdoor access contributes to slightly higher flow rates and air changes, particularly in obstruction-free conditions. This suggests that the availability of outdoor air exchange can enhance overall ventilation performance, albeit to a lesser extent than the choice of inlet configuration.

Furthermore, the simulation provides valuable insights into the relationship between volume flow rates and air changes per hour across different scenarios. It demonstrates that higher volume flow rates generally correspond to more frequent air changes. Still, this relationship is not strictly linear and can be influenced by factors such as room volume and air distribution patterns. CFD predictions emphasize the superior efficiency of plenum-based systems in ideal conditions, the significant impact of obstructions on airflow dynamics, and the potential benefits of combining different inlet types and outdoor access for optimal ventilation. The slight superiority of Scenario 2 over Scenario 1 in unobstructed conditions suggests that a combination of side and plenum inlets with open outdoor access might offer the most effective ventilation strategy.

### 3.3. The Effect of Adding Outdoor Access Without Adding Additional Airflow (Plenum Inlets)

The comparison of SO (Scenario 3) and SNO (Scenario 4) conditions, based on updated CFD simulation predictions, provides detailed insights into the performance of sidewall-based ventilation systems under varying outdoor access configurations. Both scenarios rely on sidewall inlets for airflow but differ in whether outdoor access is open (SO) or closed (SNO).

When no obstructions are present, the SO condition, with open outdoor access, achieves a volume flow rate of 10.7 m^3^/s (22.7 × 10^3^ cfm) and an air-change rate of 46.0 per hour. In contrast, the SNO condition, with closed outdoor access, produces a lower flow rate of 9.3 m^3^/s (19.6 × 10^3^ cfm) and an air-change rate of 39.8 per hour. Open outdoor access in SO enhances ventilation by allowing greater air exchange with the external environment, resulting in a 15.8% increase in airflow compared to SNO.

However, when a pen is introduced as an obstruction, the dynamics shift. The SO condition experiences a significant reduction in performance, with the flow rate dropping to 6.2 m^3^/s (13.2 × 10^3^ cfm) and air changes per hour decreasing to 26.8. Meanwhile, the SNO condition outperforms SO under these obstructed conditions, achieving a higher flow rate of 7.2 m^3^/s (15.2 × 10^3^ cfm) and an air-change rate of 30.8 per hour. This represents a 14.9% improvement in airflow for SNO compared to SO when obstructions are present.

These results highlight key trade-offs between the two configurations. The SO condition excels in unobstructed environments due to its open outdoor access, which facilitates higher air flow and ventilation efficiency. Conversely, the SNO condition demonstrates greater resilience to obstructions, likely because its closed system stabilizes airflow patterns and minimizes external disturbances that could exacerbate turbulence around barriers like pens.

The observed trade-offs between SO and SNO configurations in the ventilation performance suggest that specific operational needs should guide the addition of open-door access. For environments where maximizing ventilation under unobstructed conditions is critical, SO is preferable due to its superior efficiency with open outdoor access. On the other hand, SNO offers better performance due to its more stable internal environment for settings with frequent obstructions or where stability under varying conditions is prioritized. These findings underscore the importance of tailoring ventilation strategies to specific applications to optimize airflow and air-change efficiency under diverse conditions.

### 3.4. Comparisons with Other Computational Fluid Dynamics Studies of Poultry House

CFD studies of poultry house have consistently demonstrated that buildings with seemingly similar designs can exhibit markedly different airflow and thermal behavior when variables such as fan type, inlet geometry, bird distribution, or seasonal set-points are altered. As a result, direct numerical comparisons between previous studies and between those studies and the present work should be approached with caution. Nevertheless, a critical examination of prior research remains valuable for identifying key insights and overarching trends.

The contributions of the present study include the comprehensive validation of a CFD model specifically designed for cage-free hen houses with outdoor access that has not been previously validated. While Blanes-Vidal et al. [4] focused on conventional cross-ventilated broiler houses and Seo et al. [5] addressed natural ventilation in closed systems. This study bridges the gap by modeling the complex interactions between negative pressure systems, outdoor access, and wire-mesh pen obstructions.

Numerous authors have analyzed broiler-house tunnel ventilation. Küçüktopcu et al. [6] reported that a mechanically ventilated broiler house reached zone temperatures higher than 27 °C in summer despite high airflow. By using CFD results, a retrofitted hen house was found to have reduced peak temperature by approximately 3 °C and removed stagnant zones. Choi et al. [9] developed a transient CFD model for a 14-fan broiler house and showed that asymmetric inlet baffling can flatten those gradients but slightly sacrifices total airflow. Ma et al. [27] demonstrated that tunnel flow can over-cool birds nearest the pads while under-ventilating those at the fan end, stressing the value of spatially resolved CFD before any hardware is added.

Layer-house studies point to similar trade-offs. Chen et al. [12] developed a full-scale cage-free model with 2365 individually modeled birds. They showed that alternative mid-wall inlet and ceiling-exhaust concepts maintained hen-level temperature within comfort limits while keeping the ventilation rate in the low-temperature range in cold weather. Hu et al. [8] compared a novel positive- and negative-pressure combined system (PNCV) with conventional negative-pressure ventilation and found that PNCV increased the mean cage-zone airspeed and reduced the maximum in-house temperature differential to less than 2.9 °C. Du et al. [28] tested front-wall inlets in an aviary and found that they reduced vorticity at the windward end and mitigated the high-temperature pocket adjacent to fans without requiring additional energy input.

Cross-flow or hybrid systems can also outperform classical tunnel flow when designed carefully. Bustamante et al. [29] and Hospitaler et al. [30] modeled cross-mechanically ventilated broiler barn and demonstrated that an improved semi-tunnel configuration could achieve more uniform air flow velocities than either pure tunnel or side-wall intake designs. Key findings of referenced CFD studies are summarized in Table 5.

Taken together, these findings reinforce two conclusions that are directly relevant to the present study. First, inlet/fan positioning and control logic, not just gross ventilation rate, largely dictate airflow uniformity and thus bird-level comfort. Second, CFD models that embed realistic boundary conditions such as bird sensible and latent loads, porous-media resistance, and dynamic fan performances consistently predict both beneficial and detrimental micro-zones that on-farm spot-checks alone can miss. By incorporating a pen modeled as a porous-jump, field-measured fan performance, and scenario-specific boundary conditions, the current model aligns with practices in the literature. In other words, its prediction that wire-mesh pens impede across-pen mixing and create significant temperature gradients is therefore credible and highlights the uniqueness of our geometry.

Nevertheless, pitfalls remain for any inter-study comparison. Reported validation errors in air speed and temperature, as well as differences in mesh density, turbulence model, and simplifications of geometries, can all shift results by more than the biological response thresholds. Moreover, external climate, bird age, and stocking density strongly influence suitable ventilation scenarios.

For example, thermal requirements of a hen house are known to be age-dependent [31,32]. These age-related requirements significantly influence facility heat loads, ventilation rates, and air distribution patterns, e.g., young chicks require higher ambient temperatures and generate minimal metabolic heat. In contrast, mature birds have lower temperature requirements but produce substantially more metabolic heat due to their increased body mass and higher metabolic activity.

Commercial poultry facilities must accommodate these changing requirements through adjustable heating systems, variable ventilation rates, and potentially different air distribution strategies for different production phases. CFD modeling of such facilities would benefit from incorporating age-dependent heat generation models, variable thermal comfort zones, and growth-stage-specific behavioral patterns that influence air movement and heat distribution.

Considering that multiple factors affect optimal ventilation set-points simultaneously, it is possible that identical quantitative targets may not be appropriate across facilities. Therefore, it is recommended that design guidance emphasizes airflow patterns, velocity uniformity, and the temperature-humidity relationship of the actual conditions in the poultry house rather than absolute numeric targets, leveraging the capabilities of CFD modeling and simulation.

## 4. Conclusions

To investigate the effect of outdoor access on floor-raised hen production and welfare, one of the Penn State research rooms was equipped with configurable outdoor access. But the addition of these outdoor access features is known to disrupt conventional negative pressure ventilation performance. To mitigate this negative effect of outdoor access, overhead plenum inlets were operated to achieve either positive or neutral pressure ventilation air distribution in the research room. Due to the complexity of geometry and the numerous possible combinations of ventilation equipment, it is not straightforward to identify an optimal ventilation operation condition for a desired environment. To that end, a validated computational fluid dynamics (CFD) ventilation model is expected to make it possible to study desirable ventilation conditions under different operation conditions.

Leveraging our previous research, we developed and validated a CFD model of one of the Penn State Poultry Education and Research Center (PERC) research rooms, which included two exhaust fans, sidewall ventilation inlets, wire-meshed pens, outdoor access, and plenum inlets. Outdoor access alters airflow paths in cage-free facilities, disrupting conventional negative-pressure design. Our validated CFD model confirms that integrating overhead plenum inlets can restore control by operating under positive or neutral pressure, thereby significantly increasing air-change rates relative to sidewall-only systems in unobstructed layouts while sustaining distribution when pens obstruct flow. Although open pop-holes raise the air-exchange rate, they may elevate pathogen-entry risk, and plenum-driven positive pressure mitigates this trade-off by directing fresh air from the ceiling to the exhaust. In practice, deploying plenum-assisted ventilation can enable producers to meet welfare mandates for outdoor access without sacrificing environmental control or biosecurity. Neutral pressure emerges as an energy-efficient alternative that balances draft reduction with microclimate uniformity, albeit with greater sensitivity to wind.

The comparison between the CFD model prediction and the field measurements of air velocity and temperature showed the validation error within practical design margins, confirming the model’s suitability as a decision tool. With these findings, this study lays the groundwork for designing cost-effective, welfare-friendly hen housing environments that strike a balance between production efficiency and animal comfort and health. While our study focused on the fundamental validation of ventilation systems without birds present, practical application to commercial poultry facilities requires incorporating actual poultry house conditions, e.g., stocking density, age-dependent thermal requirements, and heat generation patterns.

## Figures and Tables

**Table 1:** Ventilation scenarios and status of exhaust fan, side wall inlets, plenum inlets, and outdoor access.

	Scenario 1	Scenario 2	Scenario 3	Scenario 4
Sidewall ventilation inlet	Closed	Open	Open	Open
Plenum ventilation fan and inlet	Open	Open	Closed	Closed
Outdoor access	Open	Open	Open	Closed
Exhaust Fan	Off	On	On	On
Acronym based on open ventilation inlets	PONE Plenum inlet Outdoor Access No Exhaust Fan	SPO Side inlet Plenum inlet Outdoor Access	SO Side inlet Outdoor Access	SNO Side inlet No Outdoor Access

**Table 2:** Measured and predicted air velocity and temperature in Pen 9 at 0.53 m, 1.13 m, and 1.73 m from the floor.

	Location	Measured Velocity (m/s)	Predicted Velocity (m/s)	Prediction Error (Based on Measurement)	Measured Temperature (°C)	Predicted Temperature (°C)	Prediction Error (Based on Measurement)
Scenario 1 (PONE)	Top	0.00	0.02	N/A	22.1	20.4	7.7%
	Middle	0.49	0.32	34.8%	21.6	20.4	5.6%
	bottom	0.07	0.09	−28.4%	21.9	20.4	7.0%
Scenario 2 (SPO)	Top	0.07	0.02	71.4%	20.3	22.0	7.5%
	Middle	0.25	0.28	−12.0%	20.3	21.4	5.2%
	bottom	0.27	0.20	26.7%	20.4	21.6	6.1%
Scenario 3 (SO)	Top	0.14	0.28	−100.2%	21.7	22.2	0.8%
	Middle	0.19	0.30	−59.5%	21.1	21.9	4.5%
	bottom	0.17	0.04	76.5%	21.5	22.4	3.5%
Scenario 4 (SNO)	Top	0.14	0.19	−35.7%	21.7	22.4	3.3%
	Middle	0.21	0.22	−3.3%	21.5	22.2	3.3%
	bottom	0.10	0.05	50.0%	21.7	22.9	5.5%

**Table 3:** Measured and predicted pressure of each ventilation scenario.

Scenario	Measured Pressure (Pa, Gauge Pressure)	Predicted Pressure (Pa, Gauge Pressure)	Prediction Error (Based on Measurement)
Scenario 1 (PONE)	0.1	0.14	40%
Scenario 2 (SPO)	0.4	2.00	400%
Scenario 3 (SO)	0.0	−0.06	N/A
Scenario 4 (SNO)	−1.0	−1.13	13%

**Table 4:** Predicted ventilation performances with and without pen.

	Location	Volume Flow Rate (m^3^/s)	Volume Flow Rate (10^3^ cfm)	Air-Change per Hour
Scenario 1 (PONE)	Without pen	30.79	65.2	132.3
	With Pen	7.56	16.0	32.5
Scenario 2 (SPO)	Without pen	31.73	67.2	136.3
	With Pen	13.01	27.6	26.8
Scenario 3 (SO)	Without pen	10.72	22.7	46.0
	With Pen	6.24	13.2	26.8
Scenario 4 (SNO)	Without pen	9.26	19.6	39.8
	With Pen	7.17	15.2	30.8

**Table 5:** Comparison of computational fluid dynamics studies of poultry house ventilation.

Study	Main Innovation	Key Findings
Present Study	Validated CFD model for hen house with outdoor access; porous-jump modeling of wire-mesh pens	Wire-mesh pens significantly impede airflow; outdoor access disrupts negative pressure systems.
Choi et al. [9]	Dynamic temperature-variation modeling	Variable inlets trade airflow for gradient control
Hu et al. [8]	Combined positive- and negative-pressure (PNCV) validation	Increase in mean cage-zone speed and more uniform temperature
Küçüktopcu et al. [6]	Comparison between summer and winter conditions	Stagnant zones removed with circulation fans, resulting in a temperature drop
Chen et al. [12]	Modeled individual-bird	Maintained bird comfort while improving air-flow distribution
Chen et al. [11]	Disease-vector containment analysis in cage-free systems	Ventilation critical for pathogen control
Bustamante et al. [29]	First field–CFD validation of tunnel-ventilated broiler house using multi-sensor isotemporal data	Tunnel flow delivered high bird-level speeds to relieve summer heat stress; CFD revealed dead-zone near inlets and over-speed near fans
Hospitaler et al. [30] (2013)	Systematic CFD validation for cross-mechanically ventilated broiler house with GLM statistics	Model matched air-flow measurements; uniform air flow in core but stagnation near inlets and fans posing high heat-stress risk
Seo et al. [5]	Optimal natural-ventilation design with diffuser and curtains	Increase in thermal uniformity with upgraded design
Blanes-Vidal et al. [4]	Comprehensive commercial poultry house validation	Bird-level air velocities often below recommended minimums in cross-ventilated houses

## Data Availability

The data supporting the conclusions of this article will be made available by the authors on request.
